# The relevance of socio-demographic and occupational variables for the assessment of work-related stress risk

**DOI:** 10.1186/1471-2458-13-1157

**Published:** 2013-12-10

**Authors:** Alessandro Marinaccio, Pierpaolo Ferrante, Marisa Corfiati, Cristina Di Tecco, Bruna M Rondinone, Michela Bonafede, Matteo Ronchetti, Benedetta Persechino, Sergio Iavicoli

**Affiliations:** 1 Italian Workers’ Compensation Authority, Research area, Occupational medicine department, Via di Fontana Candida 1, Rome 00100, Monteporzio Catone, Italy

**Keywords:** Work-related stress, Risk factors, Italy, Epidemiological survey, Management standards indicator tool

## Abstract

**Background:**

Work-related stress is widely recognized as one of the major challenges to occupational health and safety. The correlation between work-related stress risk factors and physical health outcomes is widely acknowledged. This study investigated socio-demographic and occupational variables involved in perceived risk of work-related stress.

**Methods:**

The Italian version of the Health and Safety Executive Management Standards Indicator Tool was used in a large survey to examine the relationship between work-related stress risks and workers’ demographic and occupational characteristics. Out of 8,527 questionnaires distributed among workers (from 75 organizations) 6,378 were returned compiled (74.8%); a set of mixed effects models were adopted to test single and combined effects of the variables on work-related stress risk.

**Results:**

Female workers reported lower scores on *control* and *peer support* and more negative perceptions of *relationships* and *change* at work than male workers, most of them with full-time contracts. Age, job seniority, and educational level appeared positively correlated with *control* at work, but negatively with job *demands*. Fixed-term workers had positive perceptions regarding job *demands* and *relationships,* but more difficulties about their *role* at work than permanent workers. A commuting time longer than one hour and shift work appeared to be associated with higher levels of risk factors for work-related stress (except for *role*), the latter having more negative effects, increasing with age.

**Conclusions:**

The findings suggest that the assessment and management of work-related stress risk should consider specific socio-demographic and occupational risk factors such as gender, age, educational level, job status, shift work, commuting time, job contracts.

## Background

The assessment, prevention and control of work-related stress risk are widely recognized as one of the major challenges to occupational health and safety [1]. Work-related stress is the second most common work-related health complaint among workers in the European Union (EU) and recent findings of large European surveys are consistent with a tendency to an increasing prevalence of work-related stress risks [2,3]. This might largely be due to the rapid changes in the last few years in technology and work organization, with increasing use of non-traditional employment contracts and non-standard working hours, but also to other socio-economic and demographic factors indirectly influencing the world of work, such as economic globalization, an aging workforce and the increasing numbers of women entering the workforce.

There is also ample evidence that high-risk conditions for work-related stress have a detrimental impact on workers’ health and safety, with a clear relation in particular with cardiovascular diseases [4], mental [5] and musculoskeletal disorders [6] and workers’ psychosocial well-being [7,8]. Work-related stress can also affect an organization’s productivity and competitiveness, with further impact on social and economic costs [1,9].

In the last decade several studies have provided evidence of certain key indicators of the social and occupational determinants of health [10,11]. As a consequence, there has been widespread pressure for the inclusion of those social and individual factors as determinants of workers’ health and, according to Marmot [11], of health inequalities.

Increasing importance is being attached to socio-demographic and occupational variables in the assessment and management of work-related stress risk [1,2]. In a large multicenter European study, gender differences in work-related stress perception appeared stable across occupational groups but varied according to the geographical provenance [12]. Different patterns of generation of job stress were also described in men and women [13]. An inverted U-shaped relationship was found between age and job stress [14]. Emotional workload in younger people and lack of social support in older employees were associated with a higher risk of mental health complaints [15]. Older age and longer length of service were also associated with higher levels of stress and emotional exhaustion in a group of family physicians [16]. Low occupational status and educational level were associated with higher work-related stress risk in a large sample of French workers [17]. A large U.S. survey suggested that education could mask the effects of work-related stress on health, possibly in a gender-specific way [18]. Only limited evidence was found that shift-work with nights increased the effects of work stress on some health outcomes [19]. A recent critical review reported a negative association between short-term contracts and stress, very likely reflecting the efficiency of national welfare systems [20].

Despite the growing knowledge about these issues, further investigation is needed on the integrated role of socio-demographic and occupational factors on work-related stress risk. The aim of this study was to explore the interaction effect among such characteristics and the perception of work-related stress risk, using a validated instrument. The study started from a larger research project to develop an integrated method for the assessment of work-related stress risk based on the UK Health and Safety Executive (HSE) Management Standards (MS) approach [21,22] and reflecting the EU and Italian legislative and regulatory frameworks. The HSE Indicator Tool (IT) questionnaire has been proposed to evaluate seven areas of work design corresponding to specific risk factors for work-related stress: *demands*, *control*, *managerial support*, *peer support*, *relationships*, *role*, and *change*[21-23]. After a first step in which the Italian version of this questionnaire was validated [24], a study was conducted on a sample of over 6,000 Italian workers in order to examine the relationships between the seven MS areas and a number of workers’ demographic and occupational characteristics. Specifically, the primary aim was to verify both main and the interactions effects of socio-demographic and occupational factors on the seven Indicator Tool risk factors for work-related stress and to produce explanatory hypotheses to test in the future. This analysis will also identify the socio-economic, demographic and occupational variables to be considered for the definition of homogeneous worker groups in the assessment of work-related stress, as required by national and EU legislation.

## Methods

### Participants and procedure

Data were collected from March to September 2010, administering a structured questionnaire to obtain socio-demographic information about workers and occupational factors, and the validated Italian version of the HSE MS IT [21]. A convenient sample of organizations was selected taking into account the geographical locations of companies, their sectors of activity and sizes, with the collaboration of occupational safety and health key contact experts. Since no experimental procedures were conducted on study participants, ethical approval was not required [25]. For this study, approval was obtained by the social partners of each participant organization following preliminary information briefings. Workers’ informed consent was thus obtained on the basis of specific information from their representatives. Enrolment of the workers, data collection and analysis were all done so that anonymity and privacy were ensured.

Questionnaires provided an introductory letter summarizing the study and giving contact information; they were compiled anonymously, on a voluntary basis. The contact experts distributed, collected and mailed the completed questionnaires to the Italian National Workers’ Compensation Authority (INAIL). A self-reported questionnaire was given to every worker employed in the selected companies.

The study involved 75 organizations in the public (27%) and private (73%) sectors; only one company had branch offices outside headquarters. Out of the 8,527 questionnaires administered to workers 6,378 were returned compiled (74.8% of total), the percentage of missing responses among IT items ranging from 0.89% to 2.57%. As the percentage of missing data among socio-demographic and occupational factors was substantial (more than 30%), its distribution was analyzed. For each socio-demographic and occupational variable a binary (missing/no-missing data) function was defined and a logistic model was used to test the association with all the other work-related risk factors. As no significant patterns were found, missing data were considered completely at random and the multivariate analyses were based on the caselist after listwise delection (n = 4,123). Furthermore the differences between included and excluded participants on the seven IT scales were tested.

### Instrument

The HSE MS IT is a self-administered questionnaire used as a multi-dimensional measure of work-related stress risk. It consists of 35 items and two alternative response formats: a frequency format (1 = Never, 5 = Always) and an agree format (1 = Strongly Disagree, 5 = Strongly Agree). The seven risk factors measured are: *demands* (8 items) referring to issues like workload, work patterns; *control* (6 items) reflecting how much say a person has in the way they do the work; *managerial support* (5 items) measuring encouragement and sponsorship provided by the employer; *peer support* (4 items) measuring colleagues’ encouragement and support; *relationships* (4 items) covering promoting a positive working atmosphere to avoid conflict; *role* (5 items) asking employees whether they understand their job and whether their employers ensure they do not have conflicting roles; and *change* (3 items) measuring how organizational change is managed and communicated at work. Questions for *demands* and *relationships* (negatively phrased) were reversed to conform to the other scales, with higher values indicating better performance. To verify the internal consistency of the sample the Cronbach’s alpha coefficients were calculated for the seven HSE MS IT dimensions: *demands* (Cronbach’s alpha = 0.76), *control* (0.79), *managerial support* (0.80), *peer support* (0.82), *relationships* (0.81), *role* (0.77), *change* (0.70). Full details on this instrument can be found elsewhere [24].

### Statistical analysis

The first two tables describe the statistical sample: Table 1 shows the distributions of socio-demographic and occupational variables, Table 2 the factor means and the standard deviation by gender. To investigate associations between socio-demographic and occupational variables with each IT risk-dimension, we adopted seven mixed effects models with random company-specific intercepts. These models can handle unbalanced data and correlated measures within companies and are described by the following equation:

$$Y_{i}=\left(\mathrm{fixed}\;\mathrm{effects}\right)+{\left(\mathrm{random}\;\mathrm{effects}\right)}_{i}+{\left(\mathrm{error}\right)}_{i}$$

for *i* = 1,2,…,*n* (companies).

**Table 1:** Socio-demographic and occupational factors of the workers’ sample (n = 6,378) by gender*

	**Male**		**Female**	
	**n**	**%**	**n**	**%**
**Age (yrs)**				
18-30	321	10.1	297	13.8
31-50	2,005	63.2	1,445	67.2
50+	846	26.7	408	19.0
Missing	28	-	22	-
**Marital status**				
Single	812	25.5	617	28.6
Married	2,162	67.7	1,298	60.0
Divorced/widowed	216	6.8	246	11.4
Missing	10	-	11	-
**Educational level**				
Elementary/middle	888	28.0	371	17.3
High school	1,325	41.9	886	41.2
Master/degree	955	30.1	893	41.5
Missing	32	-	22	-
**Occupational status**				
Blue-collar workers	1,304	44.2	713	34.5
Clerks/professionals	1,044	35.3	980	47.4
Manager	606	20.5	373	18.1
Missing	246	-	106	-
**Job seniority**				
0-5 yrs	665	22.4	589	28.3
6-11 yrs	706	23.8	571	27.5
12-17 yrs	540	18.2	343	16.5
>17 yrs	1,054	35.6	577	27.7
Missing	235	-	92	-
**Job contract**				
Permanent	2,711	92.8	1,795	87.7
Fixed-term	209	7.2	251	12.3
Missing	280	-	126	-
**Working hours**				
Full-time	2,858	96.4	1,502	72.5
Part-time	107	3.6	569	27.5
Missing	235	-	101	-
**Shift work**				
Day workers	2,175	68.4	1,487	69.4
Shift workers	450	14.1	384	17.9
Night workers	558	17.5	272	12.7
Missing	17	-	29	-
**Commuting time to work**				
≤ 60 minutes	1,984	78.0	1,502	77.9
> 60 minutes	561	22.0	427	22.1
Missing	655	-	243	-
**Total**	3,200		2,172	

*Gender is missing for 1,006 subjects.

**Table 2:** Factors means and standard deviations of scales by gender

	**Female**			**Male**		
	**n**	**Mean**	**Std**	**n**	**Mean**	**Std**
*Demands*	2,168	3.50	0.64	3,192	3.51	0.62
*Control*	2,168	3.50	0.75	3,192	3.50	0.82
*Managerial* s*upport*	2,168	3.67*	0.80	3,192	3.61*	0.79
*Peer support*	2,167	3.77	0.75	3,192	3.78	0.73
*Relationships*	2,168	3.90*	0.76	3,192	4.01*	0.75
*Role*	2,168	4.35	0.56	3,193	4.36	0.58
*Change*	2,150	3.36*	0.82	3,176	3.31*	0.85

*means significantly different by gender (p < 0.05).

Both random effect (company effect) and residual (error) were assumed to be normally distributed with means zero and variances respectively $\sigma_{B}^{2}$ and $\sigma_{W}^{2}$.

As dependent variables (*Y*_*i*_), we considered the mean scores (range 1–5) for the IT subscales: *demands*, *control*, *managerial support*, *peer support*, *relationships*, *role* and *change*. Fixed effects (FEs) of the models were selected through the following procedure. For each IT subscale (response variable), FEs of all the available variables were tested in a single effect model; the FEs that were significantly associated with at least one response variable were included in multivariate models and retested all together. Then the final models were obtained by excluding variables with no significant effect on any of the seven IT mean scores.

The independent variables included in the models were: gender (male, female), age (coded on three levels: 18–30, 31–50 and >50 years), educational level (coded on three levels: elementary/middle, high school and university degree), marital status (coded on three levels: single, married or divorced/widowed), occupational status (coded on three levels: blue-collar workers, clerks and managerial), job seniority (coded on four levels: 0–5, 6–11, 12–17 and >17 working years), job contract (coded on two levels: permanent and fixed-term), working hours (coded on two levels: full-time and part-time), shift work (coded on three levels: day workers, shift workers and night workers), commuting time to work and back (coded on two levels: ≤60 and >60 minutes).

The interaction effects for which the scientific literature suggested deeper discussion and that were significantly associated with at least one IT subscale were considered. Working hours*gender [26]; shiftwork*job seniority [19] and occupational status*educational level [27] interaction effects were finally included in the models.

All the analyses were done using the statistical package SAS version 9.1. The level of statistical significance was set at 0.05.

## Results

Table 1 describes the socio-demographic and occupational characteristics of the 6,378 workers by gender. The male to female ratio was 1.47. More than 60% of workers of both sexes were aged 31 to 50 years and most were married. About a third worked on shifts and about 16% had flexible work patterns (part-time and/or fixed-term work). Fixed-term job contracts and mainly part-time work were more common for women (respectively 12.3% and 27.5%) than men (7.2% and 3.6%).

Table 2 gives the descriptive statistics (mean and standard deviation) for the seven HSE IT areas. Mean scores for men ranged from 3.36 (*change*) to 4.35 (*role*), for women from 3.31 (*change*) to 4.36 (*role*). There were no significant differences by gender except for *managerial support*, *relationships* and *change*.

Women workers gave significantly lower scores on *control* and *peer support* scales and had more negative perceptions of *relationships* and *change* at work than men (Table 3), mainly among those with full-time contracts (Table 4). The oldest group (>50 years) gave a higher score for *control* than those younger than 30 years. Both married and divorced/widowed people felt job *demands* more negatively than unmarried workers, though unmarried subjects had worse scores for *control* and *role* (Table 3).

**Table 3:** Single effects of the workers’ characteristics on the seven dimensions of perceived work-related stress (multiple mixed effects model)

	***Demands***		***Control***		***Managerial support***		***Peer support***		***Relationships***		***Role***		***Change***	
**Variables**	β*	**p**	β*	**p**	β*	**p**	β*	**p**	β*	**p**	β*	**p**	β*	**p**
*Gender*														
Women		ns	-0.09	0.003		ns	-0.11	<0.001	-0.13	<0.001		ns	-0.06	0.046
Men (ref.)	0	-	0	-	0	-	0	-	0	-	0	-	0	-
*Age*														
>51 years		ns	0.10	0.048		ns		ns		ns		ns		ns
31-50 years		ns		ns		ns		ns		ns		ns		ns
18-30 years (ref)	0	-	0	-	0	-	0	-	0	-	0	-	0	-
*Marital status*														
Divorced/widowed	-0.09	0.012		ns		ns		ns		ns		ns		ns
Married	-0.05	0.034	0.06	0.027		ns		ns		ns	0.08	0.001		ns
Single (ref)	0	-	0	-	0	-	0	-	0	-	0	-	0	-
*Educational level*														
Master degree	-0.13	0.001	0.24	<0.001		ns		ns		ns		ns		ns
High school		ns	0.21	<0.001		ns		ns		ns		ns		ns
Elementary/middle (ref)	0	-	0	-	0	-	0	-	0	-	0	-	0	-
*Occupational status*														
Blue-collar	0.13	0.002	-0.51	<0.001		ns		ns	-0.14	0.004		ns	-0.22	<0.001
Clerks/professionals		ns	-0.31	<0.001		ns		ns	-0.17	<0.001		ns	-0.23	<0.001
Manager (ref)	0	-	0	-	0	-	0	-	0	-	0	-	0	-
*Job seniority*														
>17 years	-0.11	<0.001	0.09	0.015	-0.10	0.010	-0.08	0.024		ns	0.07	0.018		ns
12-17 years	-0.11	<0.001		ns	-0.14	0.001	-0.13	0.001	-0.14	<0.001		ns	-0.10	0.018
6-11 years		ns		ns	-0.09	0.014	-0.11	0.001	-0.10	0.003		ns		ns
0-5 years (ref)	0	-	0	-	0	-	0	-	0	-	0	-	0	-
*Shift work*														
Night workers	-0.09	0.005	-0.39	<0.001		ns		ns	-0.16	<0.001		ns	-0.12	0.004
Shift workers		ns	-0.22	<0.001		ns		ns	-0.10	0.004	0.08	0.003	-0.08	0.039
Day workers (ref)	0	-	0	-	0	-	0	-	0	-	0	-	0	-
*Job contract*														
Fixed-term	0.07	0.044		ns		ns		ns	0.09	0.032	-0.07	0.024		ns
Permanent (ref)	0	-	0	-	0	-	0	-	0	-	0	-	0	-
*Commuting time to work*														
≥ 60 min.	-0.09	<0.001	-0.07	0.020	-0.07	0.020	-0.12	<0.001	-0.14	<0.01		ns	-0.09	0.005
< 60 min.	0	-	0	-	0	-	0	-	0	-	0	-	0	-
*Working hours*														
Part-time		ns		ns		ns		ns		ns		ns		ns
Full-time (ref)	0	-	0	-	0	-	0	-	0	-	0	-	0	-

*The effects estimates (*β*) are reported only for significant differences.

**Table 4:** Analysis of the effect of interaction between some workers’ characteristics (multiple mixed effects model)

	***Demands***		***Control***		***Managerial support***		***Peer support***		***Relationships***		***Role***		***Change***	
**Interactive effects**	β*	**p**	β*	**p**	β*	**p**	β*	**p**	β*	**p**	β*	**p**	β*	**p**
*Among part-time*														
Women vs men		ns	-0.18	0.029		ns		ns		ns		ns		ns
*Among full-time*														
Women vs men	-0.05	0.031	-0.07	0.016		ns	-0.10	<0.001	-0.15	<0.001		ns	-0.08	0.013
*Among aged above 50 years*														
Night vs day workers		ns	-0.45	<0.001		ns		ns	-0.17	0.011		ns	-0.18	0.026
Shift vs day workers	-0.11	0.036	-0.48	<0.001	-0.23	0.001	-0.15	0.019	-0.21	0.001		ns	-0.28	<0.001
*Among aged 31–50 years*														
Night vs day workers		ns	-0.41	<0.001		ns	-0.11	0.020	-0.13	0.003		ns	-0.13	0.014
Shift vs day workers		ns	-0.15	<0.001		ns		ns		ns	0.10	0.003		ns
*Among aged 18–30 years*														
Night vs day workers	-0.20	0.005		ns		ns		ns	-0.27	0.002		ns		ns
Shift vs day workers		ns		ns		ns		ns		ns		ns		ns
*Among blue-collars*														
Master degree vs elementary/middle	-0.10	0.050	0.21	0.001		ns		ns		ns	-0.10	0.035		ns
High school vs elementary/middle		ns	0.08	0.036	-0.11	0.004		ns		ns		ns		ns
*Among clerks*														
Master degree vs elementary/middle	-0.15	0.008	0.31	<0.001	0.16	0.024		ns	0.18	0.006		ns		ns
High school vs elementary/middle		ns	0.37	<0.001	0.14	0.036		ns	0.19	0.002		ns		ns
*Among managers*														
Master degree vs elementary/middle		ns		ns		ns		ns		ns		ns		ns
High school vs elementary/middle		ns		ns		ns		ns		ns	0.33	0.002		ns

*The effects estimates (β) are reported only for significant differences.

Workers with high school or university education reported more positive perceptions of *control* but only those with a master degree had more negative perceptions of job *demands* than the less educated people. Blue-collar workers and clerks/managers scored less on *control* and reported more negative perceptions of *relationships* and the management and communication of *change*, but blue-collars perceived even lower job *demands* than managers. A positive correlation emerged between *control* and occupational status, with a mainly large difference between blue-collar workers and managers (β = -0.51) (Table 3). Blue-collar workers and clerks with university education had less positive perceptions of *demands* than those with elementary/middle education, but higher scores in *control*. Blue-collar workers with a master degree had negative perceptions of their *role* compared to others whereas managers with higher education seemed to perceive a clearer role. A high educational level played a positive role in relation to *relationships* among white-collar workers (Table 4).

There was a weak positive correlation for *role* and *control* scores and job seniority, reaching significance only in the most experienced group (>17 years in the current job). Workers with more than five years in the current job reported more negative perceptions of *demands*, *relationships*, peer and managerial *support*. Fixed-term contract workers had more positive perceptions of *demands* and *relationships* than permanent workers but felt their *role* less clearly (Table 3).

As regards commuting time, workers who spent more than an hour traveling to and from work had more negative perceptions of all the factors except *role*, than those who spent less than an hour (Table 3).

The negative effects of shift-work on all subscales except *role* was evident from the interaction with age; the scores were significantly lower for shift and/or night workers, mainly in the oldest groups (Table 4). *Control* and *change* scores were inversely related to age. Night work was significantly associated with negative perceptions of *demands* and *relationships*, particularly among the youngest workers. Those with individual missing data had more negative perceptions of the seven IT mean scores.

## Discussion

This study explored the relationship between workers’ individual characteristics and perceived risk for work-related stress measured using the Italian validated version of the HSE IT. A large cross-sectional survey of Italian companies was designed, covering a large part of the national economic sectors. The findings confirmed that both socio-demographic and occupational characteristics significantly influence work-related stress risk factors.

In compliance with Italian regulations, analysis of these characteristics contributes to the identification of “homogeneous” groups of workers for the assessment and management of work-related stress risk. The identification of particularly sensitive groups of workers, considering suitable individual and organizational aspects, is a key issue for the prevention policy recently introduced in EU and Italian legislation.

The study sample was in line with the composition of the overall Italian workforce, as compared with national statistics. In Italy in 2010 about 5% of male and 30% of female employees worked part-time and the proportions of fixed-term workers varied from 11% for men to 15% for women. The percentage of subjects doing shift-work was comparable to that obtained from EU surveys among Italian workers [3].

As our findings confirmed, women had more negative perceptions of work-related stress risk factors than men, especially the full-time workers. This could well be due to gender role differentiation at work [28]. Traditionally, women have primarily a caretaking role and bear a higher burden of family-related responsibilities than men and that can make them more likely to feel strained by their job, particularly when they have trouble balancing work and family commitments, as in the case of full-time work [26]. In fact, by rebalancing work and family commitments through part- time work, the gender difference in perceived risk for work-related stress turned out to be irrelevant. It is worth noting that welfare deficiencies, exacerbated today by economic difficulties and the financial crisis, may also contribute to increasing the work-family imbalance.

We noted that married workers of both sexes perceived higher work *demands*. This too is likely to reflect the higher burden of family responsibilities on married workers compared to unmarried ones. As reported elsewhere [29], the weight of family responsibilities might lead to a potential work-home conflict that could reduce the individual’s ability to cope with increasing demands at work. Unmarried workers perceived less autonomy in their job and greater role ambiguity than married ones. These findings are original but are very likely due to the closer social integration and acknowledgment of married people than single ones in the Italian social and cultural contexts.

A significant interaction was found between educational level and occupational status, since blue-collar workers with the highest level of education (i.e. master degree) perceived more job *demands* and role ambiguity. These findings might be explained by a phenomenon known as over-qualification or under-employment, a condition in which people have a level or type of education or skill that exceeds their job requirements [30]. The current changing nature of the world of work has resulted in increased unemployment and, in some cases, in large numbers of over-qualified workers. There is evidence of the effect of under-employment on workers’ attitudes at work and physical and psychological health [27]. Moreover, blue-collar workers with higher education may perceive job *demands* and role ambiguity more negatively also because they are likely to be assigned more demanding jobs than their colleagues on account of their education and specialization.

Job seniority showed a positive relationship with *control* and *role* scores, confirming that the more experience a person has, the greater their autonomy and role awareness in their job [31]. At the individual level, a higher job tenure might balance the burden of responsibilities at work, emerging as higher perceived job *demands*. It is also worth noting that in our sample workers with higher seniority perceived a poorer quality of *relationships* with supervisors and colleagues and less social support at work than less senior ones. While job seniority increases, employees likely become more autonomous and the perceptions of supervisor and colleagues support may decrease. Furthermore, it is like we assist to a deterioration process of the relationships at work over time, but deeper investigation should test this hypothesis. Shift-workers perceived greater job *demands*, less autonomy at work and more conflicting relationships in the workplace, in line with previous findings [32]. Working on shifts and -- even more -- working nights does call for a high degree of adaptation on the biological, social and behavioral levels [33], it can affect social relationships and lead to potentially important work-home interference [34]. However, shift-work appeared to be associated with slightly higher role clarity at work. Interpretation of these findings is complex and multifaceted, possibly including the influence on the clarity of role and the absence of potential role conflicts. As for the possible combined effects of shift-work and aging on workers’ health and wellbeing, data are still contrasting. We found an interactive effect of aging and shift-work on most work-related stress risk areas. The negative perceptions among older workers might reflect their lower tolerance of shift-work, mainly involving greater sensitivity to physical health disorders and sleep problems [35]. However, age did not seem to boost the detrimental effect of shift-work, and particularly of night work, on *relationships* and support at work, possibly because of the higher experience among elderly workers [36].

The relationship between commuting time and perceived risk for work-related stress is widely acknowledged, like the association with physical and psychological health outcomes such as musculoskeletal disorders, cardiovascular diseases, anxiety and sleep disorders [37-39]. Long commuting time to work also reduces productivity and job satisfaction, mostly through enhancement of the perception of work-related stress risk factors [40,41]. In line with the literature, our findings confirm the negative role of commuting particularly on *demands* and *control* (where the management of working hours is extremely important) and on social support and *relationships* at work. No negative influence of commuting time was found on workers’ perceptions of their role (role clarity and conflict). This is presumably due to the fact that role is more associated with aspects related to the job design than to the job schedule.

Finally, those with a fixed-term contract had more positive perceptions of *demands* and *relationships* at work but less positive perceptions of their role than permanent workers. International studies on job contracts, fixed-term work conditions and health reported contrasting findings [20]. Several studies have indeed demonstrated that other factors (e.g. welfare policies, more flexibility in organizing work) may moderate the negative consequences on workers’ health related to fixed-term employment. However, a fixed-term contract may, in some cases, make workers feel less involved in their company, with negative consequences for role clarity.

### Limitations and future perspectives

One of the main limitations relies in the high proportion of missing data on individual characteristics. As in other self-reported studies, privacy concerns and the need for anonymity can lead to a large number of missing data. In our sample, workers who did not report their individual characteristics had more negative perceptions of the seven risk areas (assessed from IT mean scores). This suggests a personal decision to omit some socio-demographic and occupational information to avoid being identified. Nevertheless, the distribution of missing data among individual characteristics was completely at random. The analysis of factors influencing missing data is beyond the scope of the present study and it could be better investigated in the future.

A second limitation relates to the self-reported nature of our data: the findings may contain some bias on account of the common method variance. However, our aim was primarily to investigate the perceptions of psychosocial risks, which by definition are aspects that only the individual can report [42]. Future studies should investigate some objective indicators besides workers’ perceptions in order to firmly anchor our findings.

The lack of data on ethnicity is another limitation and needs to be considered in the future, particularly considering the growing presence of immigrants in the Italian and overall European workforce.

## Conclusions

Our findings illustrate the importance of socio-demographic and occupational variables for work-related stress risk factors. The most susceptible groups were female workers, for whom part –time work is one way of reducing the difficulties of balancing work and family commitments, better-educated workers, especially those who are over-qualified for the jobs assigned, and shift workers, especially older ones.

Finally, commuting time was confirmed as having an effect on work-related stress risk, whereas part–time work, especially for women, and fixed-term contracts had potentially protective effects.

It may be reasonable to conclude that the assessment and management of work-related stress risk should take into account the relations with all the social, demographic, and occupational factors involved. Analysis of these variables is particularly important in the light of the European legislation on safety and health at work, concerning the obligation to assess the risk of work-related stress, because it can provide useful information as a basis for identifying specific groups of workers at risk. We would warmly suggest focusing on interventions aimed at managing work-related stress risk in these groups, such as job scheduling, transportation demand, training for less experienced workers, and strategies for reducing work-home conflict.

## Competing interests

The authors declare that they have no competing interests.

## Authors’ contributions

AM participated to conceive the study and to define its design, participated in the statistical analysis and to draft the manuscript. PF participated to define the design of the study, performed the statistical analysis and participated to draft the manuscript. MC, CDT, MB, MR participated to conceive the study and to define its design, participated in the statistical analysis and to draft the manuscript. BMR, BP participated to conceive the study and to define its design. SI conceived the study and defined its design, participated to draft the manuscript. All authors read and approved the final manuscript.

## Pre-publication history

The pre-publication history for this paper can be accessed here:

http://www.biomedcentral.com/1471-2458/13/1157/prepub

## What this paper adds

This study shows that age, sex, job seniority, commuting time, shift work, job contract and occupational status all influence work-related risk factors;

The assessment and management of work-related stress risk should consider specific socio-demographic and occupational variables;

This study helps define groups of workers at risk to whom specific interventions could be targeted for the prevention of work-related stress and individual and organizational health problems.
